# Does Video Gaming Affect Orthopaedic Skills Acquisition? A Prospective Cohort-Study

**DOI:** 10.1371/journal.pone.0110212

**Published:** 2014-10-15

**Authors:** Chetan Khatri, Kapil Sugand, Sharika Anjum, Sayinthen Vivekanantham, Kash Akhtar, Chinmay Gupte

**Affiliations:** MSk Lab, Imperial College London, Charing Cross Hospital, London, United Kingdom; Xiamen University, China

## Abstract

**Introduction:**

Previous studies have suggested that there is a positive correlation between the extent of video gaming and efficiency of surgical skill acquisition on laparoscopic and endovascular surgical simulators amongst trainees. However, the link between video gaming and orthopaedic trauma simulation remains unexamined, in particular dynamic hip screw (DHS) stimulation.

**Objective:**

To assess effect of prior video gaming experience on virtual-reality (VR) haptic-enabled DHS simulator performance.

**Methods:**

38 medical students, naïve to VR surgical simulation, were recruited and stratified relative to their video gaming exposure. Group 1 (n = 19, video-gamers) were defined as those who play more than one hour per day in the last calendar year. Group 2 (n = 19, non-gamers) were defined as those who play video games less than one hour per calendar year. Both cohorts performed five attempts on completing a VR DHS procedure and repeated the task after a week. Metrics assessed included time taken for task, simulated flouroscopy time and screw position. Median and Bonett-Price 95% confidence intervals were calculated for seven real-time objective performance metrics. Data was confirmed as non-parametric by the Kolmogorov-Smirnov test. Analysis was performed using the Mann-Whitney U test for independent data whilst the Wilcoxon signed ranked test was used for paired data. A result was deemed significant when a two-tailed p-value was less than 0.05.

**Results:**

All 38 subjects completed the study. The groups were not significantly different at baseline. After ten attempts, there was no difference between Group 1 and Group 2 in any of the metrics tested. These included time taken for task, simulated fluoroscopy time, number of retries, tip-apex distance, percentage cut-out and global score.

**Conclusion:**

Contrary to previous literature findings, there was no correlation between video gaming experience and gaining competency on a VR DHS simulator.

## Introduction

The last few decades have seen the development of commercially available video games, which have become a part of everyday social culture. Persistent advancements in both software and hardware have led to the development of increasingly realistic video simulation games. Ubiquitous access to the Internet and additional infrastructure to support higher bandwidths has facilitated co-operative play, further increasing popularity of videogames [1]. As a result, video games have been an integral part of British childhood for almost two generations with the average gamer being 30 years old [2].

Although video gaming has been associated with negative effects, including lower academic performance [3], childhood obesity [4] as well as muscular and skeletal disorders [5], [6], studies have postulated a positive correlation between video gaming and technical skill performance in laparoscopic [1], [7]–[12], endoscopic [13]–[15] and vascular surgery [16]. However, this association remains unexplored within orthopaedic surgical skills.

The formation of psychomotor mechanisms such as ‘attentional weighting’ refers to the ability to discriminate less significant aspects and give increased attention to those elements of greater importance [17]. For example, when performing a laparoscopic cholecystectomy, a surgeon with greater attentional weighting can concentrate on the operative task via a screen without being distracted by events in the background. Gaming has been shown to improve such skills.

Alongside this, developments of spatial awareness and hand-eye co-ordination are key skills that can be acquired from video gaming [8], [18]. Younger surgeons have been found to develop technical skills faster than their senior colleagues, and a crossover of skills from video gaming has been suggested as a reason [19].

### Video gaming influence in other surgical disciplines

Within orthopaedic trauma, there have been no studies conducted to observe the effects of video gaming on technical objective performance metrics. Previous studies in laparoscopic simulators have found that experience gained from 2D screens of video gaming has influenced performance by improving tasks such as object transfer and figure of eight [9]. Like laparoscopy, placement of a dynamic hip screw (DHS), one of the commonest orthopaedic trauma procedures, relies on viewing images on a 2D screen to coordinate instruments in a 3D plane.

Within the previously mentioned trial, the methodology focused on providing training on specific video games before conducting a trial [9]. However, a short period of two weeks of gaming does not truly represent a ‘gamer’ as it does not accommodate for those people who have spent long period of their life acquiring technical skills and visuo-spatial awareness from commercial video games.

### Aim

To investigate the effect of video gaming on acquisition of technical skills on a haptics-enabled, virtual-reality (VR) DHS simulator. Primary objectives included seven objective performance metrics.

### Null-hypothesis

Extent of video gaming does not correlate with psychomotor performance, as measured by objective metrics, on a VR DHS simulator.

## Methods

### Ethics

Imperial College Research Ethics Committee granted ethics for this project (MEEC1213-17). Informed, written consent was gained from all participants before the study commenced.

### Simulator Equipment

All participants were tested on TraumaVision VR (SimBones AB, Linkoping, Sweden), a haptics-enabled, VR, DHS simulator with the additional function of simulated fluoroscopy. The software runs on a standard computer desktop with two foot-pedals (to demonstrate antero-posterior (AP) and lateral fluoroscopic radiography) and a Phantom pen stylus (SensAble Technologies Inc., Massachusetts, USA) to simulate guide wire positioning, drilling, reaming and screw driving.

### Power Calculation

The power calculation was based on a preliminary pilot study using surgical trainees with the same inclusion and exclusion criteria. The pilot study consisted of ten participants naïve to simulation. Out of the objective metrics, we determined the Cohen's d effect to be 0.954 to reflect the effect size. We determined that with a two-sided p-value of 0.05 and a power of 80% (β = 0.2 with largest SD = 86.3) we required at least 38 participants in total. To compensate for possible drop-outs, we recruited 42 participants with at least 19 participants in a group.

### Participants

38 novice surgical trainees were recruited for this cohort study. Group 1 (n = 19, the ‘gamer’ group) and Group 2 (n = 19, the non-gaming group).

#### Inclusion Criteria

Naivety to DHS procedures and orthopaedic VR simulation. For the video gaming cohort, gamers were defined as a having a minimum of one hour per week of gaming on any platform, whilst non-gaming was defined as less than one hour of gaming, per calendar year.

#### Exclusion Criteria

Previous exposure to DHS procedures or orthopaedic simulation.

After informed consent, participants viewed a four-minute video to guide them through the steps of the DHS procedure. All testing was carried out in isolation.

### Operative Tasks

The standardised task consisted of the following steps:

- Place a guide wire into the femoral neck and head using the pre-selected 135 degree fixed angle guide, under fluoroscopic guidance
- Select an appropriate reamer length and ream over the guide wire
- Select and insert an appropriate length lag screw
- Insert a four-hole 135 degree plate and align this correctly to the long axis of the femur
- Reduce the plate to the lateral femoral cortex with a simulated mallet
- Drill through the distal hole of the plate through both cortices
- Insert a depth gauge and measure the width between the medial and lateral femoral cortices
- Select and insert an appropriate length screw

### Metrics assessed

Primary objectives consisted of seven objective performance metrics measured by the simulator including (i) total procedural time (secs), (ii) total fluoroscopy time (secs), (iii) number of radiographs taken (n), (iv) Tip-Apex Distance (TAD) (mm), (v) number of unique attempts or retries (n) to place the guide-wire, (vi) the probability of cut-out (%) according to Baumgaertner's graph [20], and (vii) global rating score (%), as calculated by the simulator. A unique attempt was defined as withdrawal of the guide-wire from the cortex proceeded by another attempt to drill. Groups were compared at baseline and then after their tenth (last) attempt. Learning curves using median scores for each metric per attempt were plotted on a line graph following a linear multiple regression best-fit trendline. This way both peaks and troughs could be established fluctuating with number of attempts. Trends in baselines and rate of improvement could also be outlined. Correlation coefficient for each trendline was calculated.

### Statistical Analysis

All data was recorded as median and Bonett-Price 95% confidence intervals. Data was confirmed as non-parametric by the Kolmogorov-Smirnov test. Analysis was performed using the Mann-Whitney U test for independent data whilst the Wilcoxon signed ranked test was used for paired data. A result was deemed significant when a two-tailed p-value was less than 0.05.

## Results

38 participants completed the study where both cohorts were completely naïve to orthopaedic simulation. The most commonly used systems were the PlayStation 3 (PS3), and ‘Other (PC)’, which were used equally by seven participants (36.8%). No users reported use on mobile gaming systems (iOS & Android) (Table 1).

**Table 1:** Gamer group platforms.

Platform	Primary Console Frequency	Percentage (%)
*PS3*	7	36.8%
*Xbox*	4	21.1%
*Wii*	1	5.3%
*iOS*	0	0.0%
*Android*	0	0.0%
*Other (PC)*	7	36.8%
*Total*	19	100.0%

### Baseline

Seven metrics were used to demonstrate heterogeneity and minimisation of selection bias. There was no difference between cohorts at baseline in all seven metrics (Table 2).

**Table 2:** Baseline comparison between groups of all objective metrics.

Metric	Gaming group Median (95% CI)	Non-gaming group Median (95% CI)	*p*-Value^a^
*Time (s)*	524 (392–657)	549 (421–678)	0.73
*Fluoroscopy (s)*	29.6 (4.53–54.6)	42.3 (19.5–65.2)	0.99
*Number of Radiographs*	38.0 (26.4–49.6)	43.5 (28.1–58.1)	0.39
*Number of Retries*	1.00 (0.47–1.53)	2.00 (0.42–3.58)	0.51
*Tip-Apex Distance (mm)*	25.9 (20.4–30.9)	25.4 (20.2–30.6)	0.90
*Cut-Out (%)*	4.19 (1.64–6.74)	3.32 (0.10–6.55)	0.80
*Score (%)*	47.8 (31.4–64.1)	35.1 (16.6–53.6)	0.37

a Significance determined by Mann-Whitney U Test.

### Time (seconds)

**Figure 1:**
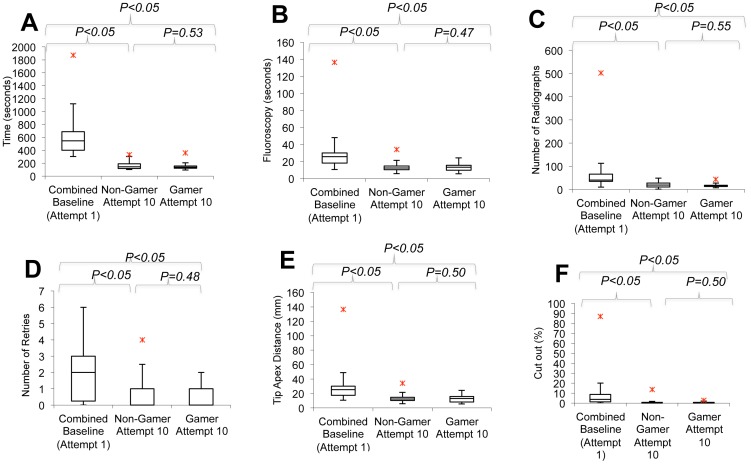
Box and Whisker Plots showing improvement in metrics in A: Time (s), B: Fluoroscopy (s), C: Number of Radiographs (n), D: Number of Retries (n), E: TAD (mm) and F: Cut-Out (%). Red stars indicate max outliers.

The gamer group took 5% less time than the non-gamer group however this result was insignificant (*p* = 0.53). Both gamer and non-gamer groups demonstrated significant results in terms of improvement via training by taking 74% and by 73% less time respectively (Figure 1A, Table 3).

**Table 3:** Comparison of metrics for training and control groups before and after training.

	Gamer Group				Control (Non-Gamer Group)				Intergroup comparison	
	*(Median +95% CI)*				*(Median +95% CI)*					
	*Attempt 1*	*Attempt 10*	*Change (%)*	*p-value* a	*Attempt 1*	*Attempt 10*	*Change (%)*	*p-value* a	*Overall Change 10^th^ vs. 10^th^ (%)*	*p-value* b
**Time (s)**	524 (392–657)	138 (120–155)	74% decrease	<0.01	549 (421–678)	146 (111–181)	73% decrease	<0.01	5% decrease	0.53
**Fluoroscopy (s)**	29.6 (4.53–54.6)	28.7(16.9–40.6)	3% decrease	0.43	42.3 (19.5–65.2)	27.5 (13.1–42.0)	55% increase	0.30	4% increase	0.47
**Number of Radiographs**	38.0 (26.4–49.6)	13.0(10.9–15.1)	66% decrease	<0.01	43.5 (28.1–58.1)	19.0(11.1–26.9)	56% decrease	<0.01	32% decrease	0.55
**Number of Retries**	1.00 (0.47–1.53)	0.00(0.00–0.53)	100% decrease	<0.01	2.00 (0.42–3.58)	0.00(0.00–0.53)	100% decrease	0.02	No change	0.48
**TAD (mm)**	25.9 (20.4–30.9)	13.1(9.92–16.3)	49% decrease	<0.01	25.4 (20.2–30.6)	12.31(10.1–14.5)	52% decrease	<0.01	6% increase	0.50
**Cut-Out (%)**	4.19 (1.64–6.74)	0.47(0.03–0.91)	89% decrease	<0.01	3.32 (0.10–6.55)	0.35(0.02–0.68)	89% decrease	<0.01	26% increase	0.50
**Score (%)**	47.8 (31.4–64.1)	95.9(91.8–100)	50% increase	<0.01	35.1 (16.6–53.6)	94.9(91.1–98.7)	63% increase	<0.01	3% increase	0.41

a Significance determined by Wilcoxon signed ranks test.

b Significance determined.

### Fluoroscopy used (seconds)

Comparing cohorts, the gamers utilized 4% more fluoroscopy than the control group but this was shown to be insignificant (*p* = 0.47). With increasing attempts in gamer and non-gamer groups, there was an insignificant increase in fluoroscopy by 3% and 4% respectively (Figure 1B, Table 3).

### Number of Radiographs

By comparison, gamers took 32% less radiographs than the non-gamer group but this was insignificant (*p* = 0.55). The gamer group showed a significant decrease of 65% in the number of radiographs taken. The control group also displayed significant results of a 56% decrease (Figure 1C).

### Number of Retries

There was zero difference in the number of retries by the end of both cohorts' tenth attempts (*p* = 0.48). Adjusting for training effect, both the gamer and non-gamer group showed a significant decrease of 100% in retries of inserting the guide-wire (Figure 1D).

### TAD (mm)

The gaming group had a 6% greater TAD compared to non-gamers (*p* = 0.50) by the end of testing. The gamer group showed a significant improvement by 46% in their TAD and similarly the non-gamer group showed a significant improvement of 52% (Figure 1E).

### Probability of Failure (%)

Between cohorts there was a 26% increase in failure rate (cut-out) for gamers, however, this was insignificant (*p* = 0.50). Both gamers and non-gamers showed a significant decrease in the probability of cut out at tenth attempt (87% and 89% respectively; Figure 1F).

### Global Score (Max: 39)

**Figure 2:**
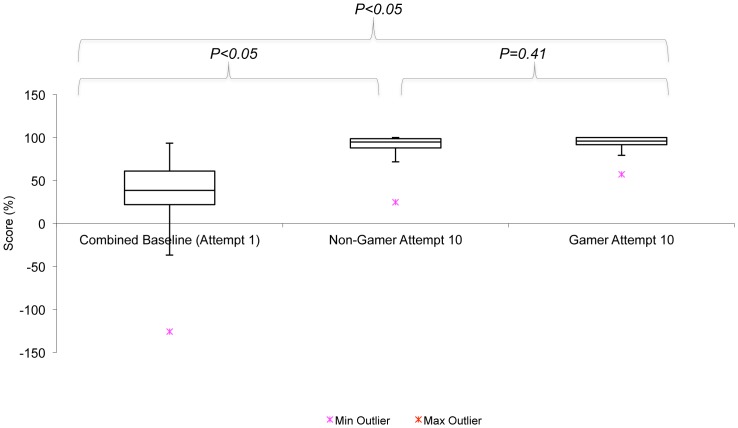
Box and whisker plot to show improving Global Score (%), comparing first (pooled) and last attempts for both cohorts.

**Figure 3:**
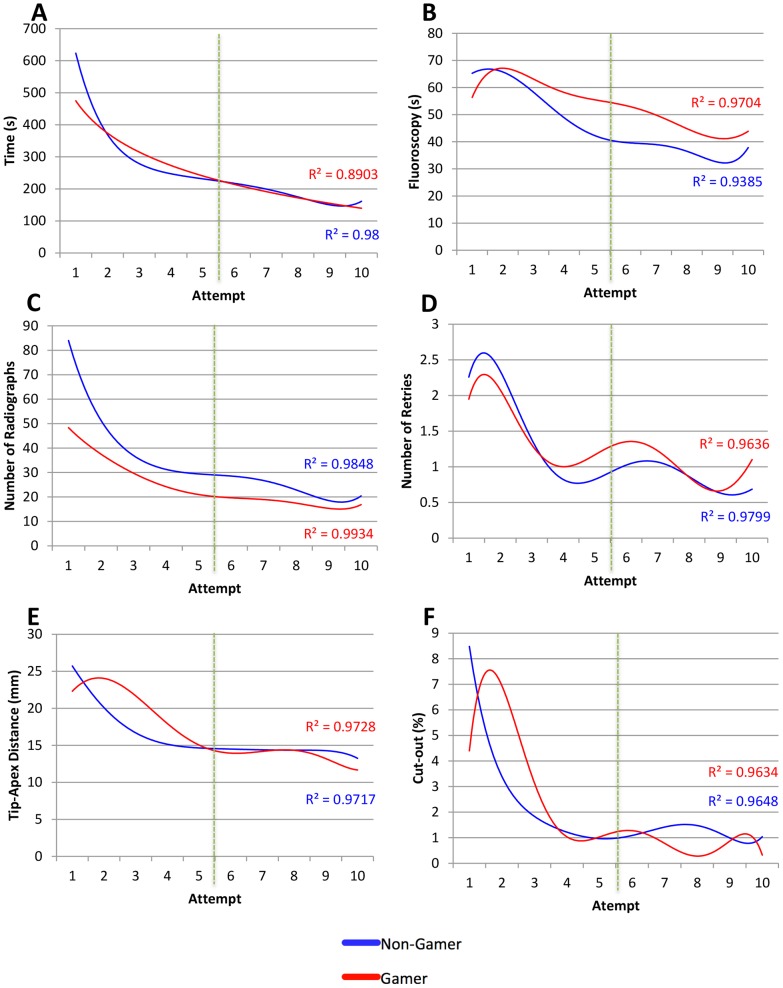
Multiple regression analysis between performance and attempt for: A: Time (s), B: Fluoroscopy (s), C: Number of Radiographs (n), D: Number of Retries (n), E: TAD (mm) and F: Cut-Out (%). Green dashed line indicates one week apart.

**Figure 4:**
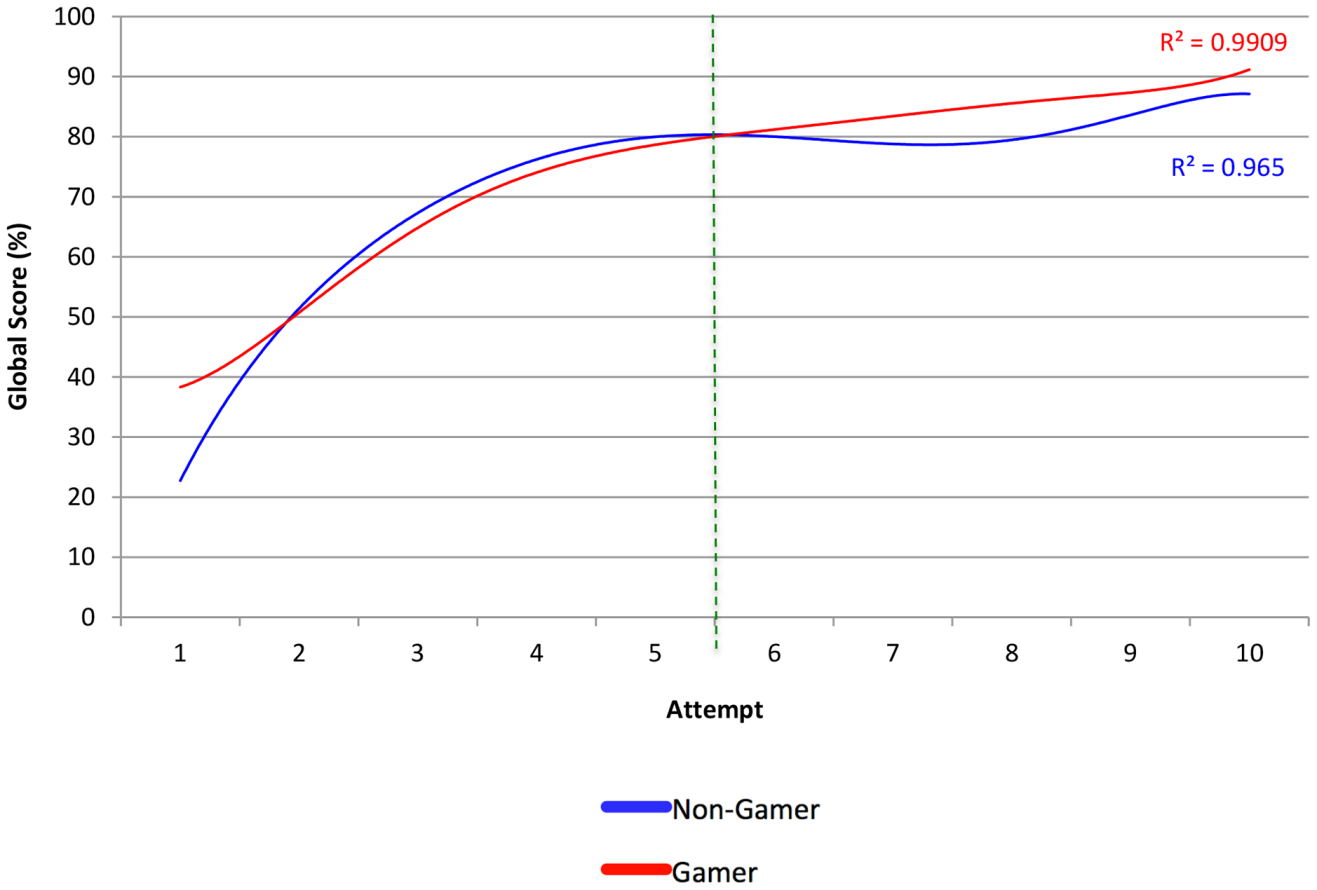
Line Graph to show improvement in performance per attempt in Global Score (%).

There was a 3% increase in the gamer's global score, compared to non-gamers but this was not significantly different (*p* = 0.41). Global score improved significantly with repeated attempts in both groups (51% - gamers, 63% - non-gamers *p<0.01*) (Figure 2). Learning curves in Figures 3 & 4 demonstrate a similar trend for both cohorts with gamers scoring a better baseline but the rate of improvement was greater in non-gamers. Both cohorts scored a similar baseline in the second week but the gamers scored higher throughout every attempt in the second week. Peak scores were also achieved by gamers and non-gamers by the last attempt.

### Summary of Results

The null hypotheses were accepted for all objective metrics. Both cohorts demonstrated a training effect as evidenced by the significant improvements in all performance metrics. However, there was no significant difference between the cohorts in any of the metrics (Table 3). This was further confirmed by plotting multiple regression trendlines of metric scores against number of attempts in Figure 3 and Figure 4. There was no difference at baseline between both cohorts. Additionally, throughout each attempt there was no difference in any metric where a cohort outperformed the other. Consequently, the learning curves were similar for every metric for both cohorts provided that any difference observed was due to variance.

## Discussion

In this study, we did not demonstrate a difference in skill acquisition between gamers and non-gamers. Although a coincidental significant training effect was observed in both cohorts (except for fluoroscopy), the video gaming cohort was not superior in any metric compared to the non-gaming group. The correlation coefficients in Figure 3 & 4 also demonstrate how precisely the polynomial regression trend lines followed the scores per metric. The commonalities between metrics included scoring a better baseline by second week and peak scores being achieved by the last attempt for both cohorts.

Video gaming results in improved spatial relationships, visual attentional capacity and enables visual multitasking [8]. Hence, exposure to video gaming should enrich aptitude for surgical skills, which requires attention to multiple skills in hand-eye coordination, manual dexterity, ambidexterity and triangulation while also accounting for non-technical skills of communication and monitoring the patient.

A study by Rosenberg *et al*. found that video gaming improved basic surgical tasks, such as knot tying, but was unable to influence more complex surgical tasks [9]. It has been suggested that there is a visuospatial advantage in gamers to perform complex surgical tasks but only after basic surgical skills are mastered [21]. A repeated study evaluating the effect of video games using more experienced orthopaedic trainees rather than naïve ones may reveal a significant difference in performance metrics.

Within the field of laparoscopic surgery, Giannotti *et al*. found that after a period of training on the Nintendo Wii, medical students performed superiorly to those without training on a laparoscopic simulator, Lap Mentor [10]. Badurdeen *et al*. associated a correlation between baseline performances on three Wii games with laparoscopic score [11]. Rather than relying on a short burst of training on a gaming console, Rosser *et al*. stratified for lifetime experience of gaming, and found that both current and past video gaming experience was associated with improved performance in laparoscopic tasks [1]. Aside from the Wii, other gaming platforms rely on either on a mouse and keyboard interface (PC), or a bimanual joystick with button combination (PS3/XBOX). With the absence of a physical object that needed to be manipulated in space (as found in the Wii), there was an additional absence of transference of visuospatial orientation, which may have been gained in the previous Wii-based studies.

Gentile *et al*. have argued that it is the form and the mechanics of the game that are of greater importance than the content or amount of video games played [22]. Hence each individual may be able to improve on only certain skills, but not significantly improve all skills following a universal trend. On comparison, our study incorporated a heterogeneous set of games, and whilst one game may have improved a certain metric for a group within the gamer cohort, the others, not exposed to this game will not have seen the same benefit.

The previous studies looking at the correlation between laparoscopic skills and extent of exposure to video gaming do not take into account other surgical simulation platforms. TraumaVision expects the participant to complete the DHS procedure with one hand using a stylus pen to be manipulated in space (somewhat similar to using a laparoscope). However, our simulation programme does not take into account path length and hand speed like the VR laparoscopic and arthroscopic simulators do. The majority of objective performance metrics looks more into mechanical factors of the surgical steps. These included lengths, angulations and visuospatial awareness on achieving optimal TAD using 2D AP and lateral fluoroscopic views.

One limitation of our study is that the DHS simulator has not undergone transfer and concurrent validity analysis to ensure that the skills assessed are relevant to the real operating theatre environment. However, the simulator measures clinically validated objective metrics, which has the potential to demonstrate transfer validity into the clinical scenario. Further work should however assess these criteria so that the non-effect of video gaming can be extrapolated to real life operating.

Our study is unique in demonstrating that video game exposure does not necessarily correlate with improved DHS simulation performance and scoring. Most importantly, there is no evidence to support that gamers will become technically better at performing DHS procedures.

## Conclusions

Contrary to previous findings in literature, there was no difference between those with extensive video gaming experience and those without in improving performance on a VR DHS simulator. The DHS simulator, unlike other popular endoscopic simulators, does not take into account the same metrics such as path length and hand speed.

## Supporting Information

Table S1 **Raw data available from the study.**
(XLSX)

## References

[1] Rosser JC, Lynch PJ, Cuddihy L, Gentile DA, Klonsky J, et al (2007) The impact of video games on training surgeons in the 21st century. Arch Surg 142: 181–6 discusssion 186. 17309970 10.1001/archsurg.142.2.181

[2] Association TES (n.d.) Industry Facts. Available: http://www.theesa.com/facts/index.asp. Accessed 23 November 2013.

[3] Gentile DA, Lynch PJ, Linder JR, Walsh DA (2004) The effects of violent video game habits on adolescent hostility, aggressive behaviors, and school performance. J Adolesc 27: 5–22. 15013257 10.1016/j.adolescence.2003.10.002

[4] Strauss RS, Knight J (2013) Influence of the Home Environment on the Development of Obesity in Children Richard S. Strauss and Judith Knight

[5] Lemons R (n.d.) Nintendo Issues Game Gloves - GameSpot. Available: http://www.gamespot.com/articles/nintendo-issues-game-gloves/1100-2541755/. Accessed 23 November 2013.

[6] Brasington R (1990) Nintendinitis. N Engl J Med 322: 1473–1474. 2330022

[7] Rosser JC, Gentile DA, Hanigan K, Danner OK (2012) The effect of video game “warm-up” on performance of laparoscopic surgery tasks. JSLS 16: 3–9. 22906322 10.4293/108680812X13291597715664 PMC3407453

[8] Green CS, Bavelier D (2003) Action video game modifies visual selective attention. Nature 423: 534–537. 12774121 10.1038/nature01647

[9] Rosenberg BH, Landsittel D, Averch TD (2005) Can video games be used to predict or improve laparoscopic skills? J Endourol 19: 372–376. 15865530 10.1089/end.2005.19.372

[10] Giannotti D, Patrizi G, Di Rocco G, Vestri AR, Semproni CP, et al (2013) Play to become a surgeon: impact of Nintendo Wii training on laparoscopic skills. PLoS One 8: e57372. 23460845 10.1371/journal.pone.0057372 PMC3583870

[11] Badurdeen S, Abdul-Samad O, Story G, Wilson C, Down S, et al.. (2010) Nintendo Wii video-gaming ability predicts laparoscopic skill. Surg Endosc 24: 1824–1828. Available:10.1007/s00464-009-0862-z 20108147

[12] Grantcharov TP, Bardram L, Funch-Jensen P, Rosenberg J (2003) Impact of hand dominance, gender, and experience with computer games on performance in virtual reality laparoscopy. Surg Endosc 17: 1082–1085. 12728373 10.1007/s00464-002-9176-0

[13] Kolga Schlickum M, Hedman L, Enochsson L, Kjellin A, Felländer-Tsai L (2008) Transfer of systematic computer game training in surgical novices on performance in virtual reality image guided surgical simulators. Stud Health Technol Inform 132: 210–215. 18391288

[14] Schlickum MK, Hedman L, Enochsson L, Kjellin A, Felländer-Tsai L (2009) Systematic video game training in surgical novices improves performance in virtual reality endoscopic surgical simulators: a prospective randomized study. World J Surg 33: 2360–2367. 19649553 10.1007/s00268-009-0151-y

[15] Van Dongen KW, Verleisdonk E-JMM, Schijven MP, Broeders IAMJ (2011) Will the Playstation generation become better endoscopic surgeons? Surg Endosc 25: 2275–2280. 21416186 10.1007/s00464-010-1548-2 PMC3116125

[16] Hislop SJ, Hsu JH, Narins CR, Gillespie BT, Jain RA, et al (2006) Simulator assessment of innate endovascular aptitude versus empirically correct performance. J Vasc Surg 43: 47–55. 16414386 10.1016/j.jvs.2005.09.035

[17] Goldstone RL (1998) Perceptual learning. Annu Rev Psychol 49: 585–612. 9496632 10.1146/annurev.psych.49.1.585

[18] Walter H, Vetter SC, Grothe J, Wunderlich AP, Hahn S, et al (2001) The neural correlates of driving. Neuroreport 12: 1763–1767. 11409755 10.1097/00001756-200106130-00049

[19] Van Hove C, Perry KA, Spight DH, Wheeler-Mcinvaille K, Diggs BS, et al (2008) Predictors of technical skill acquisition among resident trainees in a laparoscopic skills education program. World J Surg 32: 1917–1921. 18553192 10.1007/s00268-008-9643-4

[20] Baumgaertner MR, Curtin SL, Lindskog DM, Keggi JM (1995) The value of the tip-apex distance in predicting failure of fixation of peritrochanteric fractures of the hip. J Bone Joint Surg Am 77: 1058–1064. 7608228 10.2106/00004623-199507000-00012

[21] Lynch J, Aughwane P, Hammond TM (2010) Video games and surgical ability: a literature review. J Surg Educ 67: 184–189. 20630431 10.1016/j.jsurg.2010.02.010

[22] Gentile DA (2005) Violent video game effects on children and adolescents “ L. 57: 337–358. 16402007

